# A scalable platform for clinical immunophenotyping: assay design and quality control for high complexity flow cytometry

**DOI:** 10.1186/2051-1426-3-S2-P251

**Published:** 2015-11-04

**Authors:** Yoshinobu Koguchi, Tanisha Meeuwsen, Iliana Gonzalez, William Miller, Keith S Bahjat

**Affiliations:** 1 Earle A. Chiles Research Institute, Portland, OR, USA; 2 Earle A. Chiles Research Institute, Robert W. Franz Cancer Research Center, Providence Cancer Center, Portland, OR, USA

## 

In the early 1980's it was demonstrated that density gradient isolation of peripheral blood mononuclear cells led to an unpredictable loss of lymphocyte populations and unacceptable levels of error for even routine CD4+ and CD8+ T cell enumeration. The error associated with this manipulation was further amplified by errors associated with cryopreservation. Based on these results, clinical flow cytometry laboratories have uniformly adopted whole blood lysis techniques for enumeration of leukocytes in peripheral blood. We have developed an extended menu of standardized immunophenotyping assays performed using anticoagulated whole blood. Based on the recommendations of the Human Immunology Project, the assays offer results that can be correlated with other clinical sites where these recommendations are followed, while offering flexibility for characterization of additional antigens and cell populations. The “Core” assays are cocktails of 8-10 antibodies that identify the cell populations of interest. To each core, an unlimited number of additional antigens of interest can be quantified. This is accomplished by replicating the core cocktail as needed. Added to this core cocktail are antibodies for detection of inducible antigens. These inducible antigens are detected using fluorochromes and detectors optimized for sensitivity and reproducibility. Included with each assay is a process control. This control uses a stabilized whole blood preparation that allows the entire staining procedure to be evaluated, from pipetting of the blood through RBC lysis and analysis. Target values are determined internally for each lot of control material and each run is verified to fall within the acceptable range for each parameter. The laboratory also performs a standardization calibration protocol to adjust photomultiplier tube voltages based on a fluorescent calibration bead. This calibration allows for quantitative fluorescence (Median Fluorescence Intensity, or MFI) to be compared over the duration of a study. Through the use of process controls, instrument standardization, and reagent validation, day-to-day and user-to-user variability is minimized. This approach allows for the complexity of clinical research while minimizing the error typically associated with such assays in the research setting. Adoption of similar practices can improve data quality and thus, the opportunity to identify changes in peripheral immune composition related to disease state and treatment.

